# Carotid steal syndrome with recurrent syncope: A novel manifestation of Takayasu arteritis

**DOI:** 10.21542/gcsp.2025.62

**Published:** 2025-12-31

**Authors:** Jasmin Kaur Bhullar, Hiten Patel

**Affiliations:** 1 Department of Internal Medicine, University of North Carolina Health Southeastern, Lumberton, NC 28358, USA; 2 Division of Interventional Cardiology, University of North Carolina Health Southeastern, Lumberton, NC 28358, USA

## Abstract

Syncope as a manifestation of Takayasu arteritis is rare. We present a case of recurrent syncope in a 33-year-old woman. Clinical, laboratory, and imaging findings confirmed a new diagnosis of Takayasu arteritis. There was severe cerebrovascular involvement. Invasive angiography revealed vertebro-carotid artery collaterals causing a steal phenomenon. This resulted in vertebrobasilar insufficiency. To our knowledge, this is the first reported case of syncope due to carotid steal syndrome in Takayasu arteritis.

## Introduction

Takayasu arteritis (TA) with syncope is uncommon with <40 reported cases on PubMed^1^. Concurrent vertebrobasilar insufficiency (VBI), TA, and syncope are extremely rare. Thus far, the reported mechanism for VBI has always been limited to subclavian steal^2^. In subclavian steal a proximal subclavian artery stenosis reverses vertebral artery flow. Here, we report a case of carotid steal causing VBI in TA with syncope. Carotid steal syndrome is clinically and pathophysiologically distinct from subclavian steal. Unlike subclavian steal, which diverts blood from the posterior to the upper extremity circulation, carotid steal involves retrograde diversion from the vertebral system into the carotid circulation. This leads to posterior circulation hypoperfusion without preceding arm ischemia.

Carotid steal reflects a different hemodynamic burden on the vertebrobasilar system. It may explain the predominance of central neurologic symptoms in the absence of limb symptoms. Although carotid steal is a known cause for syncope^3^, it has never been reported in the context of TA. Previous literature suggests that carotid artery ultrasound has limited sensitivity for detecting distal steal mechanisms. In such cases, invasive angiography is valuable, allowing precise localization of stenoses^4^. In the present case, invasive angiography revealed carotid steal as the mechanism of VBI. It delineated the collateral pathways and precisely localized the flow reversal^5^.

## Case report

A 33-year-old Hispanic woman was referred to cardiology for evaluation of six syncopal episodes. She reported lightheadedness, dizziness, left arm pain exacerbated by movement, left-sided headaches, jaw claudication, intermittent blurry vision, and transient left-sided vision loss over the past year. Constitutional symptoms were limited to fatigue, without fever, weight loss, or night sweats. The brachial artery blood pressure in a sitting position was 60/40 mmHg on the left and 100/76 mmHg on the right, with absent left radial pulse and bruits over the left subclavian and bilateral carotid arteries. Temporal pulses were 1+ on the left and 2+ on the right.

**Figure 1.**
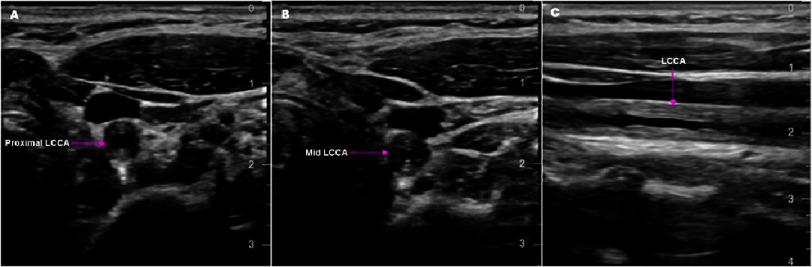
Carotid artery duplex scan. (A) Proximal left common carotid artery (LCCA) in transverse plane with stenosis (pink arrow). (B) Mid LCCA in transverse plane with stenosis (pink arrow). (C) LCCA in longitudinal plane with stenosis (pink arrow).

Carotid ultrasonography showed >75% luminal stenosis of the left common carotid artery (LCCA) (Figure 1) with antegrade flow in both vertebral arteries.

**Figure 2.**
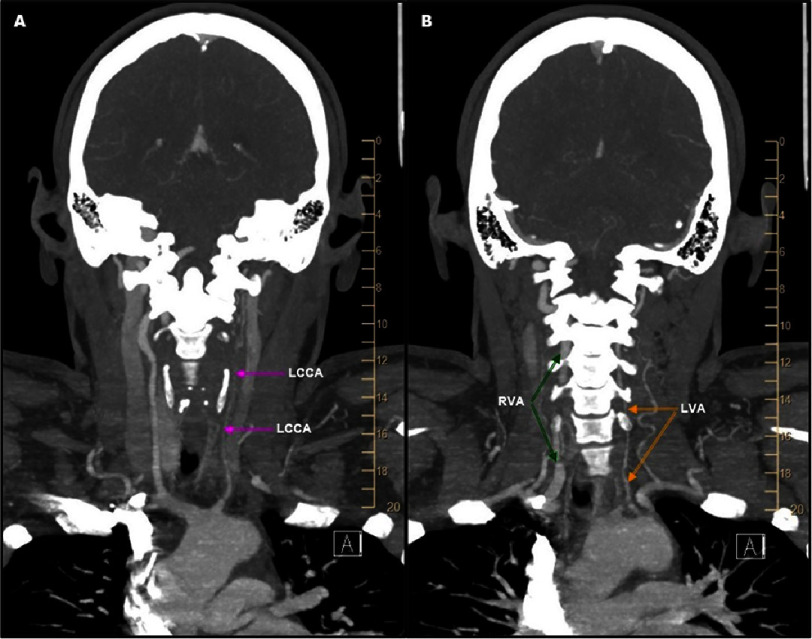
Computed tomography angiogram of head and neck. (A) LCCA with stenosis (pink arrows). (B) Hypoplastic LVA (orange arrows) and compensatory hypertrophic right vertebral artery (RVA) (dark green arrows).

Computed tomography (CT) angiography showed a string sign with distal occlusion of the LCCA (Figure 2A), a hypoplastic left vertebral artery (LVA), and a hypertrophic, patent right vertebral artery (RVA) (Figure 2B).

**Figure 3.**
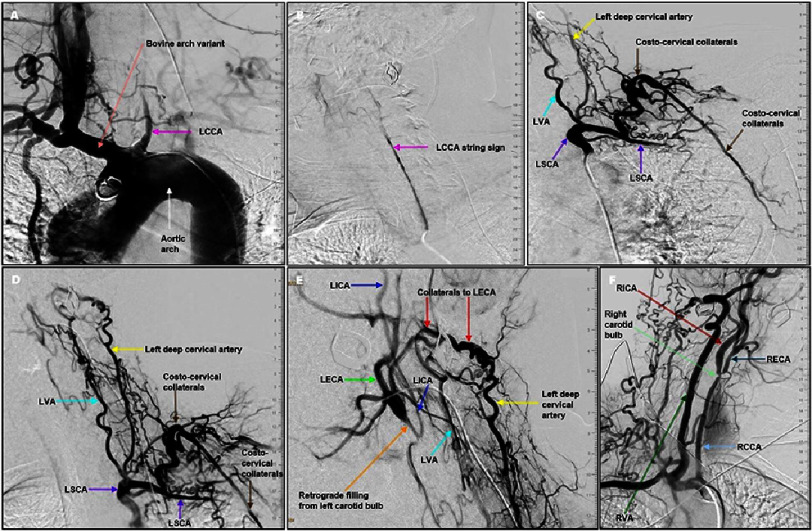
Invasive angiography. (A) Angiogram of aortic arch (white arrow) with LCCA (pink arrow) arising from bovine arch (light red arrow). (B) Selective LCCA angiography with string sign (pink arrow). (C) Selective LSCA angiography with proximal-to-mid segment patency and total distal occlusion of LSCA (purple arrows). Shows how costo-cervical collaterals (brown arrows) provide blood to left arm. It also shows hypoplastic LVA (light blue arrow) and left deep cervical artery (yellow arrow). (D) Selective LSCA angiography with LVA (cyan arrow) and left deep cervical artery (yellow arrow). It also shows costo-cervical collaterals (brown arrows). (E) The LVA (cyan arrow) and left deep cervical artery (yellow arrow) supply blood through collaterals (red arrows) to left external carotid artery (LECA) (green arrow) with a retrograde filling (orange arrow) of left carotid bulb extending into left internal carotid artery (LICA) (blue arrows). (F) Selective right innominate artery angiography shows patent right common carotid artery (RCCA) (light blue arrow). However, there is severe stenosis involving right carotid artery bulb (light green arrow), ostium of right internal carotid (RICA) (dark red arrow), and ostium of right external carotid artery (RECA) (dark blue arrow). The RVA (dark green arrow) is hypertrophic and patent.

Invasive angiography showed a variant bovine arch with subtotal distal LCCA occlusion (99–100%) (Figures 3A and 3B). The LVA and left deep cervical artery provided collaterals to the left external carotid artery (LECA) (Figure 3E). This retrograde steal flow filled the left internal carotid artery, resulting in cerebellar hypoperfusion. The left subclavian artery (LSCA) distal to the origin of the LVA was totally occluded. Left arm perfusion was preserved via costo-cervical collaterals (Figures 3C and 3D). Severe right internal (70%) and external (80–90%) carotid stenoses were also present (Figure 3F).

A PET-CT was ordered prior to initiation of treatment but was performed five weeks later. This initial and all subsequent PET-CT scans showed no hypermetabolic vascular uptake to suggest active vasculitis.

The patient’s inflammatory markers, including the erythrocyte sedimentation rate (ESR) and C-reactive protein (CRP) were within the normal range at presentation. Rheumatologic laboratory tests for vasculitis including autoantibodies (antineutrophil cytoplasmic antibody, antinuclear antibody, extractable nuclear antigen panel), complement levels, hepatitis, human immunodeficiency virus, cryoglobulins, serum and urine protein electrophoresis, urine protein/creatinine ratio, and urinalysis were all completely normal.

The patient was seen by rheumatology about five months after initial presentation. They established the diagnosis of TA and immediately initiated treatment. The patient was started on aspirin, cilostazol, and a tapering course of oral steroids. This improved symptoms but caused systemic steroid side effects, including anxiety, tremors, facial flushing, insomnia, weight gain of approximately 10 lb, and elevated blood pressure. Subcutaneous pregnancy-compatible adalimumab was later added as a steroid-sparing agent. Due to medication non-adherence, the patient experienced an acute symptom flare after she had missed her adalimumab for two doses. This was the only time when an inflammation marker was elevated during a follow-up. Laboratory testing showed a mild isolated elevation of the CRP at 1.18 mg/dL (reference range ⩽0.85 mg/dL) with a normal ESR. This prompted a switch of the pregnancy-compatible biologic to intravenous infliximab. The patient has not experienced any complications or adverse events related to biologic therapies to date. The plan is to start a non-biologic disease-modifying antirheumatic drug, ideally methotrexate, after family planning is complete.

The patient had been attempting to conceive prior to her initial presentation. At the time of diagnosis, she was advised to avoid pregnancy due to active TA. She was started on a combined estrogen–progestin oral contraceptive. Approximately six months after initiation of immunosuppressive therapy, the patient became pregnant. She was counseled that pregnancy would be high risk for both mother and fetus in the setting of active TA with dilation of the ascending aorta to four cm and significant residual large-vessel stenoses, which could predispose to hypoperfusion with circulatory changes during pregnancy. Pregnancy was terminated by dilation and curettage at six weeks’ gestation. She was subsequently counseled to use a non-estrogen-containing contraceptive method due to the increased thrombotic risk associated with TA. A levonorgestrel intrauterine device was inserted.

Currently, 22 months after the initial presentation, the patient remains on infliximab. She has been weaned off steroids and the rheumatologist has reduced the infliximab from 10 mg/kg to 7.5 mg/kg every four weeks this month. According to rheumatology, the plan is to continue an intermediate-dose regimen of 7.5 mg/kg every six weeks until the patient has been in remission for at least one year. Surveillance imaging demonstrated unchanged vascular stenoses despite immunosuppressive therapy. Clinically, the patient continues to experience severe symptoms, including dizziness, headaches, presyncope, and severe left arm claudication. Given the chronic and irreversible nature of the stenoses, surgical revascularization with a carotid artery bypass is planned. According to vascular surgery, the right carotid bifurcation represents a feasible target for revascularization to augment global cerebral perfusion. The LCCA and LICA demonstrate diminutive flow lumens from the aortic arch to the intracranial territory due to wall thickening and are not reconstructable. A vascular surgery appointment is scheduled in two months.

## Discussion

The differential diagnosis for recurrent syncope in a young woman with multiple stenotic cerebrovascular lesions includes vasculitis and fibromuscular dysplasia. The absence of autoantibodies is consistent with TA, which is typically seronegative. The normal ESR/CRP does not exclude active TA. Studies show that up to 20–30% of patients in the active phase of TA may have normal ESR or CRP values^6^. In this case, the diagnosis was established by the vascular surgeons and the rheumatologist based on the demographic features (young age, female sex), clinical symptoms (limb claudication, dizziness, syncope, visual changes), absence of atherosclerosis risk factors (no smoking, diabetes, hypertension, obesity, family history, or hyperlipidemia), asymmetric brachial artery blood pressures, diminished upper-extremity pulses, bruits on examination, and angiographic evidence of multifocal large-vessel stenoses. The present case met the following 2022 American College of Rheumatology (ACR)/European Alliance of Associations for Rheumatology (EULAR) criteria for TA: Age ⩽60 years at time of diagnosis, evidence of large-vessel vasculitis on imaging, female sex, arm or leg claudication, bruit over a large artery, reduced upper-extremity pulse, systolic blood pressure difference between arms ⩾ 20 mmHg, number of affected arterial territories ⩾ 3, and symmetric involvement of paired arteries. The total score was 14 out of 22 points. According to the 2022 ACR/EULAR criteria, a score ⩾ 5 points classifies a patient as having TA. These criteria demonstrate a sensitivity of 93% and a specificity of 99%^7^.

This case underscores the limitations of carotid ultrasound in evaluating syncope. Routine Doppler studies failed to detect flow reversal in the LVA. This is explained by the localization of the stenoses, as revealed by invasive angiography. Doppler measurements of the vertebral arteries are usually performed in the lower neck. In this patient, the LSCA was occluded distal instead of proximal to the origin of the LVA. This precluded a proximal LVA steal phenomenon. VBI arose from steal flow in the distal LVA. It involved diversion of blood from the LVA to the LECA at a higher cervical level. Scanning the LVA higher up in the neck (distal to the left-sided vertebro-carotid collaterals) would have demonstrated the expected reversal of blood flow in the LVA^4^. The LVA in this case was hypoplastic. A hypoplastic LVA is most often congenital. Congenital hypoplasia arises from developmental variation during embryogenesis and may be associated with other vascular variants, such as a bovine aortic arch. Differential considerations for a hypoplastic-appearing LVA include atherosclerotic disease, vasculitis-induced stenosis, fibromuscular dysplasia, chronic thrombotic occlusion, or extrinsic compression. In this case, the uniformly small caliber of the LVA along its entire course, the hypertrophic contralateral RVA, and the presence of a bovine aortic arch variant favor a congenital hypoplastic LVA. This contrasts with stenoses of the LCCA, LSCA, right carotid artery bulb, RICA and RECA, which are classic sites for inflammatory stenosis in TA. Histopathological analysis can theoretically distinguish congenital hypoplasia from acquired stenosis. However, biopsy of deep vessels such as the vertebral artery is rarely feasible.

The first PET-CT was obtained five weeks after treatment initiation. Previous meta-analyses have reported a sensitivity of approximately 80–90% for detecting active TA using PET-CT^8^. However, in this case, the scan was performed after the initiation of high-dose steroids, which can suppress metabolic activity and yield negative results. This case highlights the importance of performing PET-CT early, before the initiation of high-dose immunosuppression, as delayed imaging may obscure disease activity and lead to prolonged treatment for presumed active TA.

This case demonstrates the value of invasive angiography in complex vascular disorders. It enabled detection of vertebro-carotid collaterals as an anatomical variation and revealed a novel carotid steal mechanism as the cause for syncope in TA.

Carotid steal represents a hemodynamically and clinically distinct entity from classic subclavian steal. In classic subclavian steal, proximal subclavian artery stenosis causes reversal of flow in the proximal segment of the ipsilateral vertebral artery to perfuse the arm. This is typically preceded by arm claudication and manifests as blood pressure and pulse asymmetry. Cerebral ischemia in subclavian steal arises during episodes of increased arm use with significant arm ischemia. These patients can have collaterals from the thyrocervical and costocervical trunks to the subclavian artery branches.

By contrast, in carotid steal, retrograde flow occurs from the vertebrobasilar system toward the carotid circulation. This typically involves vertebro–external carotid collaterals that fill the internal carotid artery. This mechanism causes posterior circulation hypoperfusion directly, even without significant arm ischemia. The carotid bed steals from the vertebrobasilar circulation. Even though the carotid steal pathway via vertebro-carotid collaterals is anatomically constant, the steal effect is physiologically dynamic. Flow reversal magnitude fluctuates based on systemic, cerebral, and vascular factors. Syncope occurs only when the degree of flow diversion and perfusion pressure reach a critical threshold for vertebrobasilar hypoperfusion. The key determinants of the amount of steal flow involve complex mechanisms of cerebral perfusion balance.

Physiologically blood flow increases to regions of heightened neuronal activity through local vasodilation (e.g., during cognitive tasks, emotional stress, visual stimulation). The use-dependent flow reversal is determined by increased cerebral demand, not limb activity.

In carotid steal, the pressure gradient driving retrograde vertebro-carotid flow depends on moment-to-moment differences in perfusion pressure between the carotid and vertebrobasilar systems. The vertebro-carotid collateral network itself may dynamically open or close depending on small hemodynamic shifts. Transient changes such as systemic hypotension, exertion, dehydration, anemia, hypoxia, or neck position may accentuate this gradient, causing dizziness or syncope.

Normally, the vertebrobasilar system compensates via autoregulatory mechanisms through vasodilation or contralateral vertebral flow augmentation. However, in chronic disease these reserves can become exhausted. In the present case of vertebral hypoplasia, further compensatory mechanisms were likely limited. This predisposed to VBI independent of subclavian flow.

Previous studies have categorized the carotid steal patterns on Doppler ultrasound as “latent”, “transient”, and “continuous”^9^. In this case, complete occlusion of the LCCA created a low-pressure target vascular bed. The large size of the well-developed collaterals supported ongoing steal flow. The concomitant vertebral hypoplasia suggests that vertebrobasilar autoregulation was already maximized. The combination of these findings supports a continuous steal flow along the collaterals. However, Doppler studies to assess the flow pattern in these vessels have not been performed.

Immunosuppressive therapy was initiated in accordance with joint recommendations from rheumatology and vascular surgery. The rheumatologist noted that inflammatory markers can be normal in TA and recommended a positron emission tomography (PET) scan to identify areas of active inflammation. However, urgent PET-CT was not feasible and was scheduled for a later date. Based on angiography alone, it was unclear whether the vascular stenoses reflected active inflammation or chronic fibrotic changes. The vascular surgeon requested a trial of aggressive immunosuppressive therapy to help determine whether the patient’s symptoms were attributable to active vasculitis or fixed mechanical stenoses. This approach was likely based on prior studies demonstrating that immunosuppressive therapy can reverse vascular stenoses in a minority of patients with TA^10,11^.

Given the progressive nature and clinical severity of the patient’s symptoms, the rheumatologist advised initiating high-dose steroids along with adalimumab before the PET-CT results were available. Steroids were initiated to rapidly suppress vascular inflammation, whereas the biologic agent adalimumab takes weeks to months to achieve full efficacy. The first PET-CT, performed five weeks after treatment initiation, was negative. Despite the negative PET-CT, the rheumatologist recommended continuing aggressive immunosuppressive therapy as the patient’s symptoms worsened during prednisone tapering.

The patient’s symptoms worsened during prednisone tapering due to initial non-adherence to biologic immunosuppressants. Social determinants—including lack of insurance, financial hardship, unemployment due to disabling TA symptoms, limited English proficiency, poor social support, and transportation barriers—contributed. She missed two months of adalimumab after the initial prescription and subsequent refills due to living two hours from the clinic and pharmacy and needing costly rideshares. Even with subsequent adherence to immunosuppressants, they did not provide sufficient relief in this case. This underlines the difficulty differentiating active vasculitis from chronic vascular stenosis. Validated algorithms for this are lacking^12,13^.

Although vascular surgery evaluated the patient four months after initial presentation, there was a significant delay between diagnosis and planned revascularization. This delay was multifactorial. The vascular surgeons requested invasive angiography in addition to CT angiography and completion of rheumatologic imaging, including a PET-CT, which could not be obtained urgently and was scheduled five weeks later. They also preferred a trial of aggressive immunosuppressive therapy, reserving surgery for cases unresponsive to medical management. Surgery was further postponed when the patient experienced an acute disease flare after missing multiple doses of adalimumab, as revascularization was deferred in the setting of active inflammation. This is likely based on previous studies, which have indicated that the use of high-dose steroids, biologic immunosuppressants, and active inflammation at the time of revascularizations is associated with an increased likelihood of complications^14,15^. Medical management and surveillance are currently being continued. The patient has been weaned off steroids and the rheumatologist has reduced infliximab from high-dose to intermediate-dose this month.

Antiplatelet therapy was initiated. Interestingly, the 2018 EULAR guidelines for large-vessel vasculitis no longer recommend routine antiplatelet, whereas the 2021 ACR guidelines still do^16,17^. Further randomized trials are needed to evaluate this.

Understanding the distinction between carotid and subclavian steal is critical for selecting appropriate imaging, surveillance methods, and planning management. Routine Doppler examinations of the vertebral artery at the lower neck may miss distal flow reversals, as occurred in this case. Additional Doppler ultrasound of the carotid arteries and cranial vertebral artery may be required to detect the steal mechanisms and categorize them as latent, transient, or continuous. Detection of carotid steal may require invasive angiography to visualize the vertebro-carotid collaterals. Surveillance strategies differ between subclavian steal and carotid steal. In subclavian steal, upper limb claudication and blood pressure asymmetry can be used to monitor subclavian artery patency. In contrast, carotid steal requires imaging with Doppler ultrasound or angiography and close monitoring of neurologic symptoms, such as dizziness or syncope, rather than relying on arm claudication. Severe cerebral hypoperfusion can occur even in the absence of critical limb ischemia. Treatment strategies also differ. In carotid steal, revascularization targets the carotid pathways rather than the subclavian artery, as is done in subclavian steal.

Unfortunately, no guidelines or solid evidence have clearly defined the optimal treatment strategy for patients with TA requiring cardiovascular surgery^15^. This case highlights the importance of early, cohesive planning and close communication within a multidisciplinary team—including cardiology, rheumatology, vascular surgery, and neurology—with a shared approach to required diagnostics, the duration of immunosuppressive therapy trials, and revascularization planning.

## What have we learned?

Recurrent syncope may reflect rare systemic conditions such as TA, especially in young females. Multimodal imaging is essential. However, determining whether symptoms are caused by active vasculitis or chronic stenosis remains challenging. In longstanding disease, immunosuppressants can fail, and revascularization is usually required for chronic, irreversible stenoses.

## Limitations

This report describes a single clinical case, which limits the generalizability of its findings. Additionally, histopathologic confirmation of TA was not obtained, and the diagnosis was based on clinical, laboratory, and imaging criteria.

The subject gave written informed consent for the publication. Institutional Review Board (IRB) approval was not required for this case report.

## Acknowledgements

The authors thank Dr. Tanuja Rajan and Dr. Poornima Vinod for their contributions to the preliminary abstract and poster presentation of this case.
